# Supplementary material for: Spectral Analysis of Light-Adapted Electroretinograms in Neurodevelopmental Disorders: Classification with Machine Learning
Source: Bioengineering (Basel). 2024 Dec 28;12(1):15. doi: 10.3390/bioengineering12010015 (PMC11761560; doi:10.3390/bioengineering12010015)
Supplement: Supplementary file 1 [file bioengineering-12-00015-s001.zip › bioengineering-3364459-supplementary.pdf]

Supplementary Material

Spectral analysis of the light-adapted electroretinogram in neurodevelopmental disorders: Classification with machine learning

Paul A. Constable, Javier O. Pinzon-Arenas, Luis Roberto Mercado Diaz, Irene O. Lee, Fernando Marmolejo-Ramos, Lynne Loh, Aleksei Zhadanov, Mikhail Kulyabin, Marek Brabec, David H. Skuse, Dorothy A. Thompson, Hugo Posada-Quintero

Table of Contents

Supplementary Material 1
- Flash Strengths 2
- Training Model Parameters 3
- Medication Effects 3
- Time Domain Features........ 4
- Time domain features between sites 5

Flash Strengths

Table S1 provides the flash strengths used in the studies with conversion to log photopic cd.s.m^{-2} based on a 6 mm diameter pupil.

Td.s	log photopic cd.s.m^{-2}	Background (cd.m^{-2})	Study
12	-0.37	40	1
21	-0.12	40	1
35	0.11	40	1
70	0.40	40	1
85	0.48	30	1
113	0.60	40	1 and 2
178	0.80	40	1
251	0.95	40	1
356	1.11	40	1
446	1.20	40	1 and 2

Table S1. Flash strengths used in the two studies in Td.s and log photopic cd.s.m^{-2} based on a 6 mm pupil. The 85 Td.s is the ISCEV standard 3 cd.s.m^{-2} flash and was performed last in study 1 after the 9 randomized flash strengths. In study 2 the 113 and 446 Td.s flash strengths were used with the 113 Td.s recorded first.

Training Model Parameters

Table S2 presents the parameters used in the machine learning models for this study.

Table S2. Database parameters used for the training of machine learning models.

**Database Parameters**		
**Site**	1 = Flinders	
	2 = UCL	
	3 = Both	
**Flash Strength (Td.s)**	12, 21, 35, 70, 85, 113, 178, 251, 356, 446	
**Eye**	R = Right eye	
	L = Left eye	
Feature combination		
**Features**	TD+VFCDM	
	TD+DWT	
	TD+VFCDM+DWT	
	Selected Features	
**Flash Strength/Eye Concatenation**	One Flash Strength / One Eye	
	Same Flash Strength / Different Eye	Right-446 / Left-446
		Right-113 / Left-113
	Flash_str1, Flash_str2 / Same eye – Diff. Eye	Left-113 / Left-446
		Left-113 / Right-446
		Right-113 / Left-446
		Right-113 / Right-446
**Feature Selection (Only for the Top-1)**	None	
	Feature Importance	
	Shapley Analysis	

Table S2 Summary of test parameters used in the machine learning models. (Flash_str1 = Flash strength 1; TD = Time Domain, DWT = Discrete Wavelet Transform, VFCDM= Variable Frequency Complex Demodulation).

Medication Effects

Methylphenidate results in an increase in dopamine in the cerebral cortex and when dopamine is reduced, such as the case in Parkinson's Disease the amplitude of the ERG is also reduced. For this reason, we excluded those participants that had used any CNS targeted medication from the analysis and included only those that were medication naïve. Figure S1 illustrates the effect of 18 mg of methylphenidate on the b-wave amplitude in an ADHD participant AD10. The blue trace represents the ERG after 30 hours of no medication and the orange trace the ERG 3 hours after talking 18 mg of slow-release methylphenidate (Concerta-XL). The flash strength was 85 Td.s which is equivalent to 3 log phot cd.s.m^{-2}.

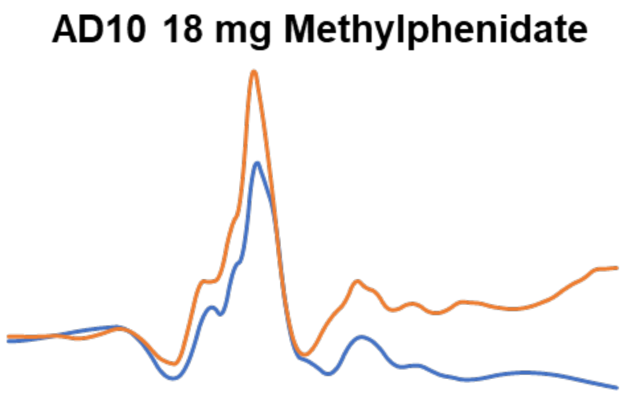

Figure S1. The amplitude of the b-wave is elevated following methylphenidate use in an ADHD participant (orange trace) compared to the blue trace recorded before methylphenidate use and following a 30 hour wash out period.

Time Domain Features

During the classification using the AdaB model the influence of Tb (time to b-wave peak) was the sole feature used (Figure S2). Consequently, a revaluation of the classification was performed excluding all Time Domain features and using only features from DWT and/or VFCDM analysis.

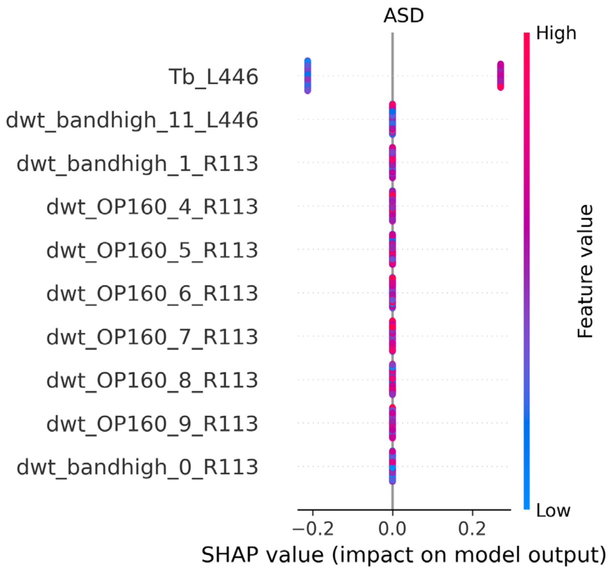

Figure S2. Shapley values for ASD classification for an AdaB model. The b-wave time (Tb) was the only featured taken by the model to perform the classification, showing a clear overfitting over this feature.

Time domain features between sites

With respect to the two sites, the features exhibit notable differences, with Flinders exhibiting a greater dispersion of the data in most of the cases. The main variability between the sites was in the amplitude of the b-wave for the ADHD participants. At UCL the b-wave amplitudes were larger than controls whilst at Flinders the ADHD participant b-wave amplitudes were lower than controls. This was in part due to the difference in the diagnostic steps taken at each site with a more stringent diagnosis accorded to those participants from UCL that is a tertiary referral site. The a-wave time derived from the ERG waveform obtained from the right eye with 113 Td.s, approximately 25% of the Flinders ASD and ADHD subjects ranged between 8.5 and 10.8 milliseconds, whereas for UCL, this interval was condensed to a range of 9.9 to 10.8 milliseconds. See Figure S3.

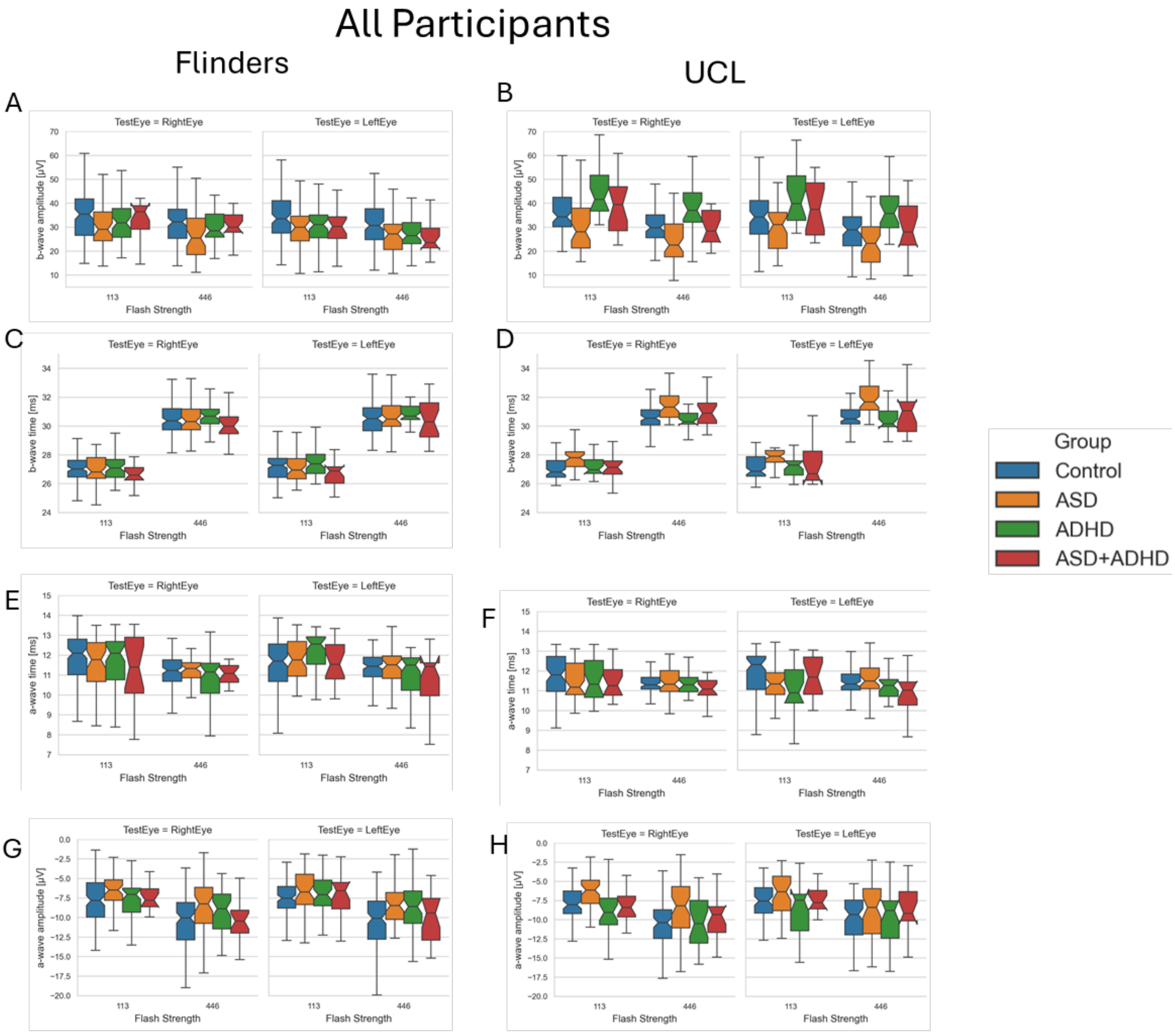

Figure S3. Distribution of the Time Domain parameters between sites for the right and left eye for all participants. For the b-wave amplitude the participants from UCL exhibited higher values (S3A and S3B). Note also the larger spread of the a-wave time values obtained in the Flinders Dataset (S3E) compared to the UCL (S3F) dataset.

When excluding the medicated subjects, the data still exhibited considerable variability, particularly within the ADHD and ASD+ADHD groups (Figure S4). The b-wave amplitudes were lower at the Flinders site compared to the UCL site for the ADHD group, and the time of the a-wave showed greater variability in the ASD+ADHD group.

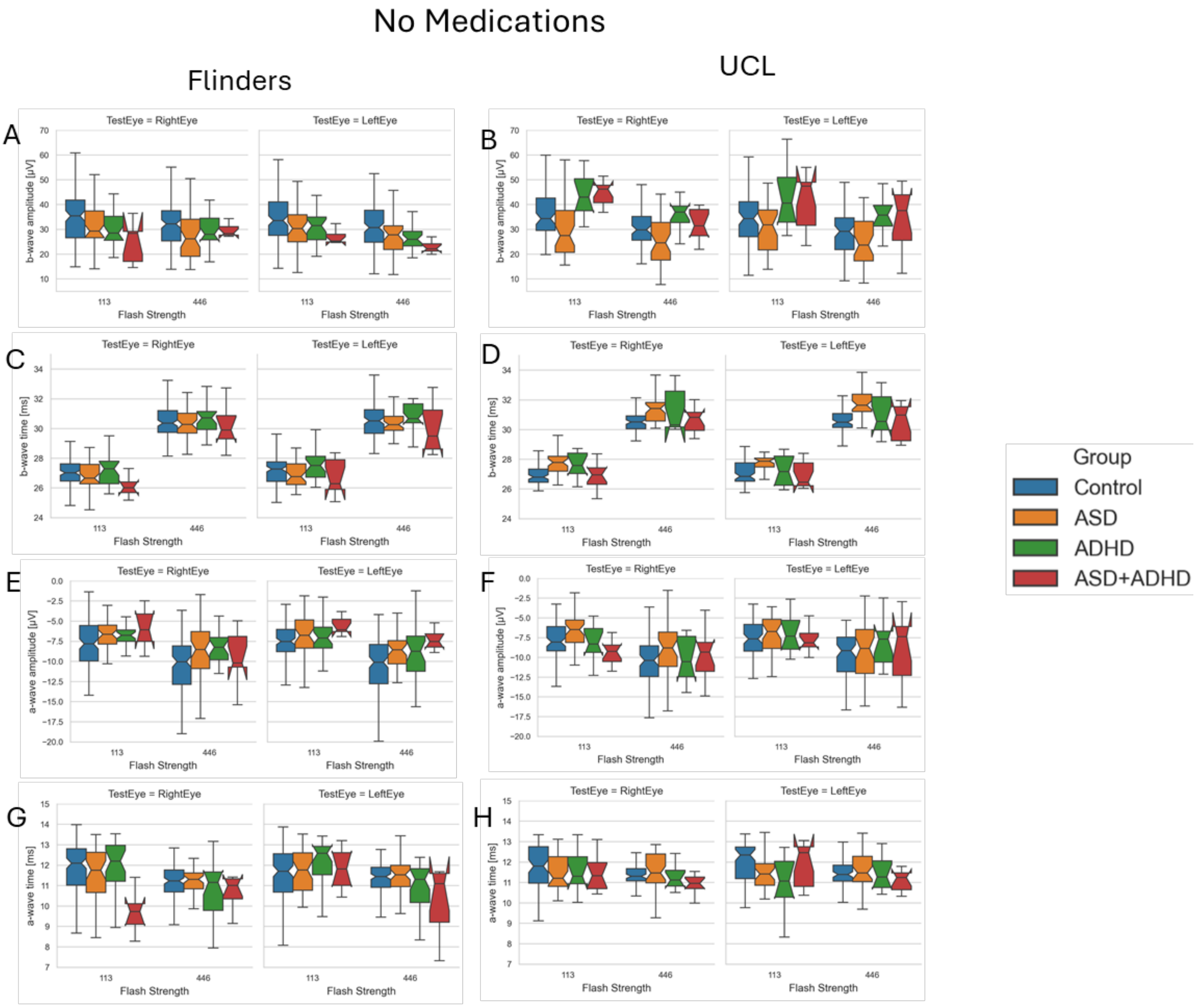

Figure S4. Distribution of the time domain parameters from each site in the participants that were medication naïve. For the b-wave amplitude the participants from UCL exhibited higher values (S4A, SB). Note also the larger spread of the a-wave time values obtained in the Flinders Dataset (S4E) compared to the UCL (S4F) dataset for the ASD+ADHD groups.

To determine the potential influence of site and the strength of clinical diagnosis with those participants at seen at UCL having a more stringent diagnostic classification. Figure S5 depicts the confusion matrices for the four classification approaches. In the 2-group (ASD) classification (Figure S5A), the XGB classifier was trained with

UCL and subsequently tested on Flinders. In this instance, only 30% of the ASD subjects were correctly classified. In the case of the ADHD classification, the RF classifier was trained with Flinders and tested on UCL, but all subjects with ADHD were misclassified as Control, as shown in Figure S5B. A comparable situation occurred with the 3-group and 4-group classification, wherein approximately 50% of ASD subjects were correctly classified, whereas the ADHD and ASD+ADHD groups exhibited extremely low or even 0% correct classifications, as shown in Figure S5C and Figure S5D. This suggests that the ERG waveforms from one site to another may not be consistent.

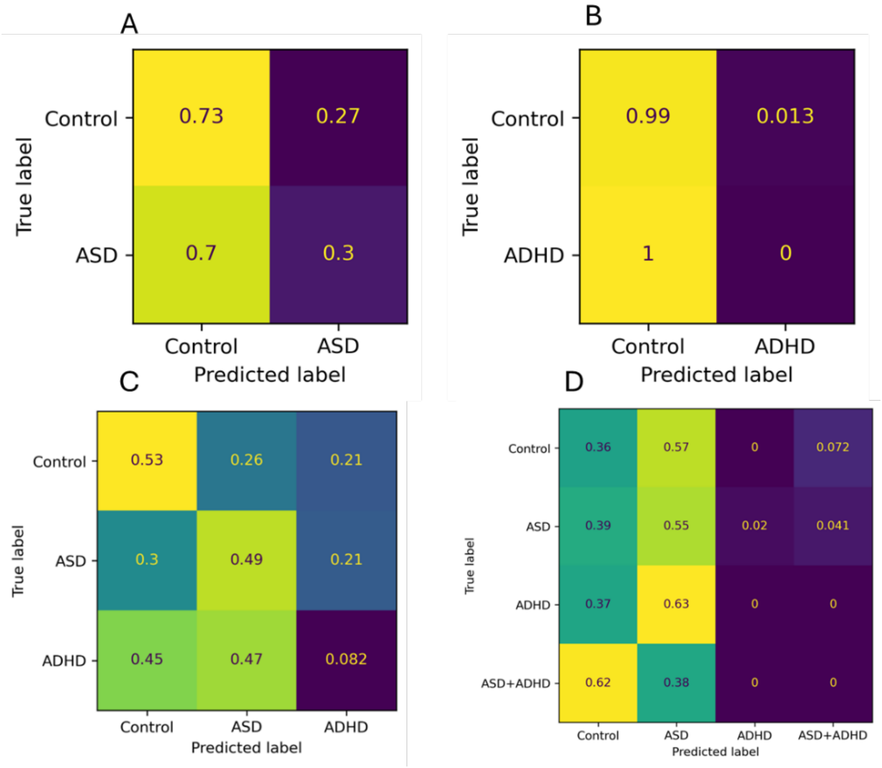

Figure S5. Confusion matrices of the best models using the site where they achieved the higher performance as training set, and the other site as testing set. (S5A) 2-group (ASD): XGB trained with UCL (site 2) and tested on Flinders (site 1). (S5B) 2-group (ADHD): RF trained with Flinders and tested on UCL. (S5C) 3-group: KNN trained with UCL and tested on Flinders. (S5D) 4-group: RF trained with Flinders and tested on UCL.

Considering the tests, which excluded medicated subjects and were conducted at a distinct site from that where the model was trained, it can be inferred that discrepancies may exist in the ERG waveforms, particularly regarding the sites in question.
